# A Partial Volume Effect Correction Tailored for ^18^F-FDG-PET Oncological Studies

**DOI:** 10.1155/2013/780458

**Published:** 2013-09-19

**Authors:** F. Gallivanone, C. Canevari, L. Gianolli, C. Salvatore, P. A. Della Rosa, M. C. Gilardi, I. Castiglioni

**Affiliations:** ^1^IBFM-CNR, Via F.lli Cervi 93, 20090 Segrate, Milan, Italy; ^2^H San Raffaele, Via Olgettina 62, 20090 Segrate, Milan, Italy; ^3^University of Milan-Bicocca, Milan, Italy

## Abstract

We have developed, optimized, and validated a method for partial volume effect (PVE) correction of oncological lesions in positron emission tomography (PET) clinical studies, based on recovery coefficients (RC) and on PET measurements of lesion-to-background ratio (*L/B*_*m*_) and of lesion metabolic volume. An operator-independent technique, based on an optimised threshold of the maximum lesion uptake, allows to define an isocontour around the lesion on PET images in order to measure both lesion radioactivity uptake and lesion metabolic volume. RC are experimentally derived from PET measurements of hot spheres in hot background, miming oncological lesions. RC were obtained as a function of PET measured sphere-to-background ratio and PET measured sphere metabolic volume, both resulting from the threshold-isocontour technique. PVE correction of lesions of a diameter ranging from 10 mm to 40 mm and for measured *L/B*_*m*_ from 2 to 30 was performed using measured RC curves tailored at answering the need to quantify a large variety of real oncological lesions by means of PET. Validation of the PVE correction method resulted to be accurate (>89%) in clinical realistic conditions for lesion diameter > 1 cm, recovering >76% of radioactivity for lesion diameter < 1 cm. Results from patient studies showed that the proposed PVE correction method is suitable and feasible and has an impact on a clinical environment.

## 1. Introduction

Molecular imaging by positron emission tomography (PET) and ^18^F-fluorodeoxyglucose (^18^F-FDG) radiotracer is currently the most commonly used method for the detection and metabolic characterisation of several oncological pathologies, given the possibility to detect foci with an increased ^18^F-FDG metabolism as those characterising tumour cells (e.g., [1, 2]). 

In the PET clinical environment, diagnosis and tumor staging are commonly assessed by qualitative visual inspection of ^18^F-FDG PET images [3–5]. Nevertheless, a quantitative analysis of ^18^F-FDG uptake in oncological lesions has been proven to be useful to differentiate benign and malignant tissues (e.g., [6]), to assess response to therapy [7–9], and to predict tumour aggressiveness [10–13].

Despite these benefits, a quantitative approach for the evaluation of PET oncological studies is not a common practice in clinical routine due to the presence of partial volume effect (PVE) on the PET images. PVE is a physical limitation resulting from the poor spatial resolution of PET systems (4-5 mm) that strongly affects the estimation of radioactivity concentration within structures less than two or three times the PET spatial resolution [14, 15].

Several techniques have been advanced to compensate for PVE in PET [15–20]. Among all PVE correction methods, more common ones are based on multiplicative numerical factors (recovery coefficients, RC), recovering the local radioactivity concentration within any small structure which uptakes ^18^F-FDG. RC can be derived from PET experimental measurements of small radioactive objects in a priori known object-to-background radioactivity concentration ratio. 

PET experimental measurements of RC have been carried out by using ^18^F-FDG radioactive spheres (hot spheres) [14]. RC coefficients were obtained as the ratio between PET measured-and-actual radioactivity concentration within the hot spheres. This approach was applied to the PVE correction of PET oncological lesions in real patients [21], since radioactive spheres were considered suitable to simulate metabolic active oncological lesions. Unfortunately, the method was able to compensate only for the spread out (spill out) of lesion ^18^F-FDG uptake into the surrounding background of the patient body not accounting for the spread in (spill in) of the background into the lesion, as it occurs in the body tissues surrounding oncological lesions in a real scenario. 

More realistic models were developed by combining RC derived from hot spots in cold background, RC from cold spots in hot background, and RC from hot spots in warm background, allowing both spill out and spill in effects to be accounted for (e.g., [15]), but were never applied to real clinical studies. 

In all cases, the applicability of RC-based PVE correction methods to PET real oncological studies is still constrained by two problems: the impossibility to estimate both the actual lesion-to-background ratio (*L*/*B*) and the actual lesion volume of oncological lesions [22–25]. For instance, measured PET images result intrinsically affected by PVE, and no a priori known information about actual *L*/*B* is available for in vivo patient studies. Furthermore, the estimation of the actual volume of an oncological lesion is one of the most debated issues in both the nuclear medicine and radiology community even though it has been coped with from different perspectives.

An RC-based PVE correction method devoted to oncological studies which overcomes the need to actually determine *L*/*B* was proposed by Srinivas et al. [26]. They performed PET measurements of hot spheres in hot background and obtained RC as a function of measured *L*/*B* (*L*/*B*_*m*_), derived from the maximum value of lesion uptake. However, RC curves were obtained as a function of the actual lesion volume of the hot spheres representing a strong limit imposed by the need to know the actual volume of lesions. As Srinivas et al. suggest, when lesion density is different from the density of the surrounding tissues, a CT study in the region of interest can provide lesion anatomical volume. Current generation multimodal computerized tomography (CT)-PET systems allow to obtain anatomical volume of a lesion temporally and spatially coregistered with the metabolic volume. Unfortunately, a lesion is not always visible on CT images and often CT anatomical volume and PET metabolic volume can deviate [27–30]. 

The applicability of RC-based PVE correction method to real oncological PET-CT images needs an estimation of *L*/*B* from measured data. Therefore, another limit of RC-based PVE correction methods is that the accuracy of the chosen RC depends on the accuracy of the technique used for the measurements of the lesion uptake [31]. For instance, operator-dependent techniques for sphere uptake measurements [24, 32–35] can induce operator-dependent differences in the estimation of RC [16]. On the other hand, operator-independent techniques [36–39] are more sensitive to the noise level of PET images and require optimisation strategies and accurate validation [16].

The aim of this work was the development of a method for PVE correction tailored for clinical application to PET-CT oncological studies. Our method is based on RC curves as functions of PET *L*/*B*_*m*_ and of PET measured lesion volume, both estimated by an operator-independent technique. The proposed PVE correction method was assessed on both anthropomorphic phantoms and in clinical ^18^F-FDG PET-CT studies.

## 2. Materials and Methods

### 2.1. ^18^F-FDG PET Studies

^18^F was produced by a cyclotron (RDS Eclipse, Siemens Healthcare) with a fixed proton beam of 11 MeV. ^18^F-FDG synthesis was obtained by nucleophilic substitution in acidic medium and subsequent purification.

A dose measurement system (Dose calibrator Pet Dose, Comecer) provided measurements of the amount of ^18^F-FDG radioactivity (administered and residual) for all phantoms and patient studies.

The multimodal PET-CT system (Discovery STE, General Electric Medical System), cross-calibrated with the dose measurement system, was used for PET-CT measurements. D-STE is a 3D hybrid system that combines a 16 multislice helical CT scanner with a PET scanner of 280 bismuth oxygen germinate crystals (4.7 × 6.3 × 30 mm^3^) arranged in 24 rings. Transaxial field of view is 60 cm and 50 cm for PET and CT, respectively. Axial field of view is 15.7 cm for PET. 

Oncological protocol was set as follows: a SCOUT scan at 40 mA, followed by a CT scan at 140 mV and 150 mA (10 sec), and 3D PET scans (2.5 min/scan) for adjacent bed positions. For each bed position, CT data were reconstructed into a 512 × 512 × 47 matrix with a voxel size of 0.97 × 0.97 × 3.27 mm^3^ [40]. For each bed position, PET data were sampled into a 128 × 128 × 47 matrix with a voxel size of 4.7 × 4.7 × 3.27 mm^3^ and reconstructed using a 3D ordered subset expectation maximization algorithm (OSEM) with corrections for random, scatter, and attenuation incorporated into the iterative process.

### 2.2. Synthetic Oncological Lesions

Perspex spheres of different diameters were used to simulate oncological lesions. 

Six spheres (diameter = 10 mm, 13 mm, 17 mm, 23 mm, 29 mm, and 37 mm) within an elliptical perspex cylinder (*d*_1_ = 24 cm, *d*_2_ = 30 cm, and *h* = 21 cm) [41] were used for the estimation of RC.

Three spheres (diameter = 9.8, 12.3, and 15.6 mm) were placed in different regions of different anthropomorphic phantoms (thorax, breast, and brain) and were used for the validation of the proposed PVE correction method in clinical-like oncological studies. Specifically, the three spheres were placed in

1. a thorax-like phantom (*d*_1_ = 20 cm, *d*_2_ = 30 cm, and *h* = 21 cm) with two cork parts simulating lungs and a cardiac insert;
2. a breast-like phantom consisting into the previously described thorax phantom (no cardiac insert) and into two plastic containers (cylinder equivalent radius = 3 cm, *h* = 10 cm) miming breasts;
3. brain-like phantom: the Hoffman 3D brain phantom [15].

Three additional nonspherical lesions consisting of zeolites were considered. Zeolites are porous aluminosilicate minerals already used to simulate oncological lesions in anthropomorphic phantoms assessed by ^18^F-FDG PET-CT studies. When soaked into an aqueous solution of ^18^F-FDG, they are able to absorb and not release ^18^F-FDG molecule, in a nonhomogeneous way for short soaking duration and in a homogeneous way for long soaking duration [42]. Zeolites with nonspherical shape and sphere-equivalent diameters = 10.3 mm, 9.9 mm, and 7.9 mm were placed in the breast phantom and were used for the estimation of bias induced by the proposed PVE correction method specifically for nonspherical and nonuniform lesions. In particular, we simulated one lesion with nonspherical shape and uniform uptake and two lesions with nonspherical shape and nonuniform uptake.

### 2.3. Patients

One hundred and thirty-seven oncological patients (46 males, 91 females, age: 28–86 years) were considered, requiring diagnostic investigation involving small lesions (diameter < 4 cm) in different body districts. 

All patients signed informed consent. They fasted for twelve hours before the PET-CT exam. ^18^F-FDG administered dose was prepared based on patient weight considering an amount of 37 MBq for each 10 kg. Administered and residual radioactivity concentrations, administration time, and patient body weight were recorded for each PET-CT study. 

108 patients underwent one basal ^18^F-FDG PET-CT study for tumor staging purpose and they were subjected to radical therapy (surgical intervention or radical radiotherapy); 29 patients underwent two ^18^F-FDG PET-CT studies, before and after receiving chemotherapy, for therapy monitoring purpose. All PET-CT studies were performed according to the oncological protocol (Section 2.1) and started 60 minutes after the injection. A total of 149 oncological lesions were assessed by ^18^F-FDG PET-CT images (49 lesions in gastric and gastro-oesophageal regions, 40 lesions in breast, 19 lesions in head and neck regions, and 42 lesions in skeleton).

Histological and therapy-outcome data were considered. Histological data were obtained from surgical intervention of 89 patients, for example, tumour histotype. In particular, for the gastric and gastro-oesophageal lesions, two histotypes were considered: signet ring cell (SRC) carcinaoma and squamous cell (SC) carcinaoma. For the breast lesions, proliferation cell index MiB-1 was provided. Disease-free survival (DFS) data at 24 months after therapy were obtained for 19 patients with cancer in the head and neck regions and treated with radical radiotherapy. 

Table 1 describes the characteristics and the available data of the considered patients.

### 2.4. The PVE Correction Method

The PVE correction method is based on recovery coefficients (RC) derived from PET measured hot-lesion-to-hot-background ratio (*L*/*B*_*m*_) and PET measured lesion metabolic volume of the six spheres within the elliptical perspex cylinder. 

*L*/*B*_*m*_ is obtained by the ratio between the PET measured sphere uptake and the PET measured background surrounding the sphere, resulting from the average over several circular regions of interest (4) around the lesion. 

RC are plotted as a function of *L*/*B*_*m*_ and of PET measured sphere metabolic volume.

The proposed PVE correction method acts at a regional level and compensates the lesion uptake underestimation on PET clinical images due to PVE by multiplying it by a proper factor (*F*) defined as *F* = 1/RC.

For each lesion detected on the PET clinical images of an oncological patient, *F* is assigned based upon the PET measured *L*/*B*_*m*_ and the PET measured lesion metabolic volume.

The PET measured sphere uptake, the PET measured sphere metabolic volume, the PET measured lesion uptake, and the PET measured lesion metabolic volume are all obtained by the an operator-independent technique described as follows.

### 2.5. The Operator-Independent Technique

An operator-independent technique was developed allowing to obtain an isocontour on that PET image including the maximum lesion/sphere uptake. The isocontour is defined at a definite threshold of the maximum lesion/sphere uptake. Such isocontour defines either the region of interest for the PET measurement of sphere/lesion uptake or the circle-equivalent section of a PET measured sphere/lesion spherical metabolic volume (isocontour volume).

The threshold is chosen by an optimisation procedure such that the PET measured metabolic volumes of spheres match their actual metabolic volumes.

### 2.6. Optimization of the Operator-Independent Technique

PET-CT independent measurements with the six spheres and the PET-CT DSTE scanner were performed according to the oncological protocol (Section 2.1) using an acquisition time of 30 min/scan in order to minimize the noise level on the PET images and considering 2 PET scans at 2 adjacent bed positions (phantom *h* = 20 cm). 

For each independent measurement, the spheres were filled with different radioactivity concentrations of ^18^F-FDG and dipped into the elliptical cylinder filled with a radioactivity concentration of ^18^F-FDG of 0.01258 MBq × mL^−1^ (background). 

PET measured metabolic volumes were calculated on the PET images according to the described operator-independent technique for thresholds at 50, 60, 70, and 80% of the maximum sphere uptake. The percentage differences between the actual sphere diameter and the derived sphere diameter were calculated using a different threshold from each PET measured volume.

The optimal threshold was chosen as the threshold giving the lowest positive percentage differences. This procedure warrants the actual sphere metabolic volume to be represented by the PET measured volume in the best possible way and at the same time allows to exclude background components.

### 2.7. RC Estimation

PET-CT independent measurements with the six spheres and the PET-CT DSTE scanner were performed as in Section 2.1.

Sphere and background radioactivity concentration obtained with the dose measurement system was regarded as the gold standard (GS), namely the best estimate of the actual radioactivity concentration. *L*/*B*_GS_ ranged from 4 to 35 (*B*_GS_ concentration from 0.0018 MBq × mL^−1^ to 0.024 MBq × mL^−1^).

As a representative example, Table 2 shows the GS radioactivity concentrations in the spheres (*C*_GS-sphere_) and in the background (*C*_GS-background_) and the derived *L*/*B*_GS_ for one of the measurements.

For all the independent PET-CT measurements, *L*/*B*_*m*_ was calculated according to the operator-independent technique at the optimal threshold. 

RC were calculated as the ratio between *L*/*B*_*m*_ and *L*/*B*_GS_. 

RC curves were obtained by combining RC values as a function of *L*/*B*_*m*_ and of sphere “isocontour” diameter. 

The RC curves were fitted using a three-parameter hyperbolic function. 

### 2.8. Validation of the PVE Correction Method

#### 2.8.1. RC Noise Sensitiveness

The sensitiveness to noise level on the PET images of the method to estimate RC was assessed.

PET-CT measurements were performed with the six spheres with *L*/*B*_GS_ ranging from 7 to 10, following oncological protocol but using different acquisition times (2.5 min, 5 min, 10 min, 15 min, and 30 min). 

For each sphere, RC was calculated at each acquisition time, and percentage differences of RC over time were obtained.

#### 2.8.2. Residual Errors after PVE Correction

The accuracy of the PVE correction method was assessed by evaluating residual errors after PVE correction. 

PET-CT measurements were performed with the three synthetic spherical lesions and with the three synthetic zeolites within the anthropomorphic phantoms, and background was filled with different concentrations of ^18^F-FDG. *L*/*B*_GS_ ranged from 4 to 35 for 31 independent experiments (*B*_GS_ concentration from 0.005 MBq × mL^−1^ to 0.0012 MBq × mL^−1^). 

Zeolites were prepared as described in [42]. They were soaked into an aqueous solution of ^18^F-FDG with an actual radioactivity concentration of 0.17 MBq × mL^−1^. One zeolite was soaked for 15 minutes to simulate a nonspherical but homogeneous tumor. The other two zeolites were soaked only for 5 seconds to simulate nonspherical heterogenous tumors. 

Zeolite weights (dry weight before soaking and wet weight after soaking) were measured by means of an analytic balance. Absorbed radioactive solution volume was estimated as the difference between wet and dry weights. Zeolite volume was measured using Archimedes' principle. Radioactivity within zeolites was calculated as radioactivity concentration of the ^18^F-FDG soaking solution multiplied by the absorbed radioactive solution weight. Radioactivity concentration within each zeolite was calculated as the ratio between radioactivity within zeolite and zeolite volume. Sphere-equivalent diameters were obtained from zeolite volumes.

For each phantom lesion (both spheres and zeolites), lesion optimised “isocontour” volume and *L*/*B*_*m*_ were measured on PET images. 

The PVE-corrected radioactivity concentration within spheres was obtained by multiplying the measured PVE-affected radioactivity concentration by the proper *F* = 1/RC. 

Percentage residual errors, as the differences between the GS and PVE-corrected radioactivity concentration, were calculated.

### 2.9. Feasibility of the PVE Correction Method

Feasibility of the PVE correction method was assessed by applying the PVE correction to the PET-CT studies of the selected oncological patients. 

Qualitative and quantitative assessment was performed under the guide of one expert nuclear medicine physician. Body-weighted standardized uptake value (SUV) was provided and calculated as the tissue radioactivity concentration corrected for the injected activity and body weight of the patient [32]. SUV quantification with PVE correction was performed for all considered lesions (149). During the measurement of *L*/*B*_*m*_, for each considered lesion, the nuclear medicine physician was informed not to include any adjacent high uptake organ in the background measurement.

Statistical correlation analysis was performed between SUV (with and without PVE correction) and the histological and therapy outcome data available for the 108 patients subjected to radical therapy.

For the 29 patients subjected to chemotherapy, the EORTC classification of response to treatment was provided [43].

Table 3 briefly describes the kind of analysis performed for the patient groups.

## 3. Results

### 3.1. Optimization of the Operator-Independent Technique

Table 4 shows, for the PET measurements of the six spheres, the percentage differences (%) between the actual sphere diameter (*d*) and the sphere diameter derived from “isocontour” volumes at 50, 60, 70, and 80%, averaged over *L*/*B*_*m*_. 

The optimal threshold giving the lowest positive percentage difference was found to be the threshold at 60%. This value represents a well compromise between a good sample of the lesion actual volume and a good sample of the lesion uptake, minimizing the possibility to include radioactivity background in the sample. Indeed a 50% threshold for the 10 mm sphere gives a negative difference between the actual sphere diameter and the sphere diameter derived from the “isocontour” volume, estimating a lesion volume that is larger than the true volume, thus bringing to include nontumour tissues adjacent to the lesion.

### 3.2. RC Estimation

**Figure 1:**
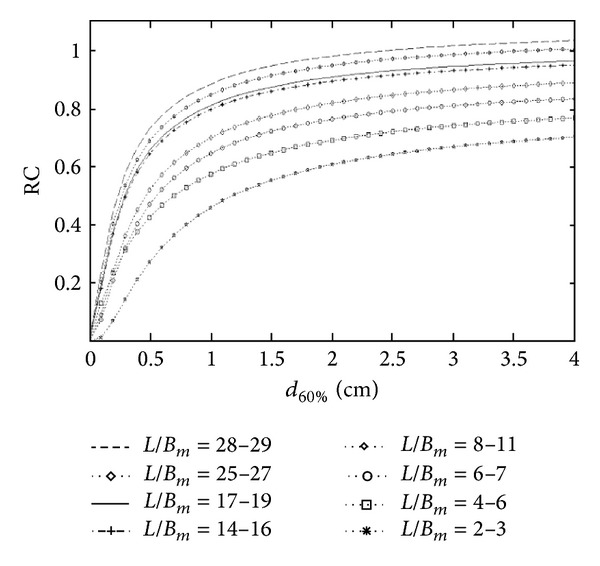
RC curves, threshold = 60%.

Figure 1 shows, for the six spheres, RC curves (8) obtained for *L*/*B*_*m*_ from 2 to 29, with sphere “isocontour” diameter derived from the optimal threshold (60%) up to 4 cm. The fit was accurate (*r* square > 0.93) for all RC curves. 

**Figure 2:**
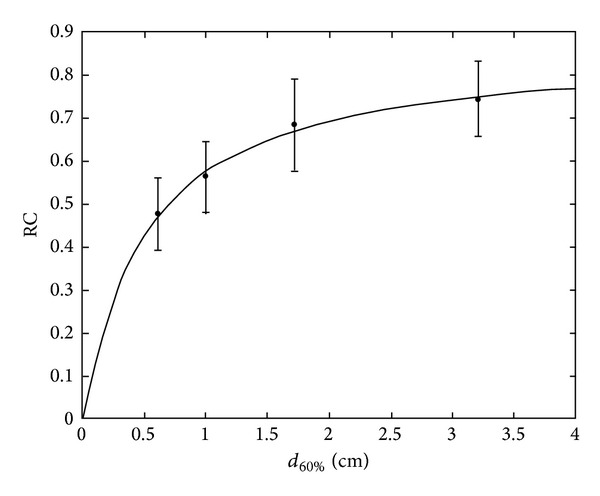
RC curves, threshold = 60%, *L*/*B*_*m*_ = 3.

Figure 2 shows, for sphere measurements, RC, error bar, and fitting curve for *L*/*B*_*m*_ = 3. The accuracy of the fit can be observed also qualitatively. 

Results show that the underestimation of radiotracer uptake due to PVE ranged from 26% up to 70% for the sphere of 10 mm diameter, from −3% up to 32% for the sphere of 37 mm diameter, and from 30 to 2 for *L*/*B*_*m*_, respectively. This confirms the severity of the error and the need for PVE correction.

Table 5 shows the percentage differences between the GS (*C*_GS-sphere_) and measured (*C*_60%_) radioactivity concentrations for the six spheres (one representative PET-CT measurement). *C*_GS-sphere_, *C*_60%_, and *L*/*B*_*m*_ are also presented. 

### 3.3. Validation of the PVE Correction Method

#### 3.3.1. RC Noise Sensitiveness

**Figure 3:**
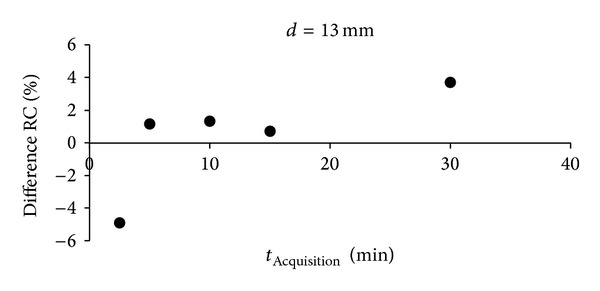
Percentage difference of RC over acquisition time.

Figure 3 shows, for the sphere with *d* = 13 mm, the percentage difference of RC over the acquisition time (2.5, 5, 10, 15, and 30 min). 

RC was found poorly sensitive to the noise level on the PET images for acquisition times in the order of 30 min down to 2.5 min (percentage difference < 5%), proving the noise independency of the method to estimate RC. This guarantees the feasibility of our RC-based PVE correction method for clinical studies of acquisition time from 2.5 min (standard whole-body PET scan/bed) up to 30 min. 

#### 3.3.2. Residual Errors after PVE Correction

**Figure 4:**
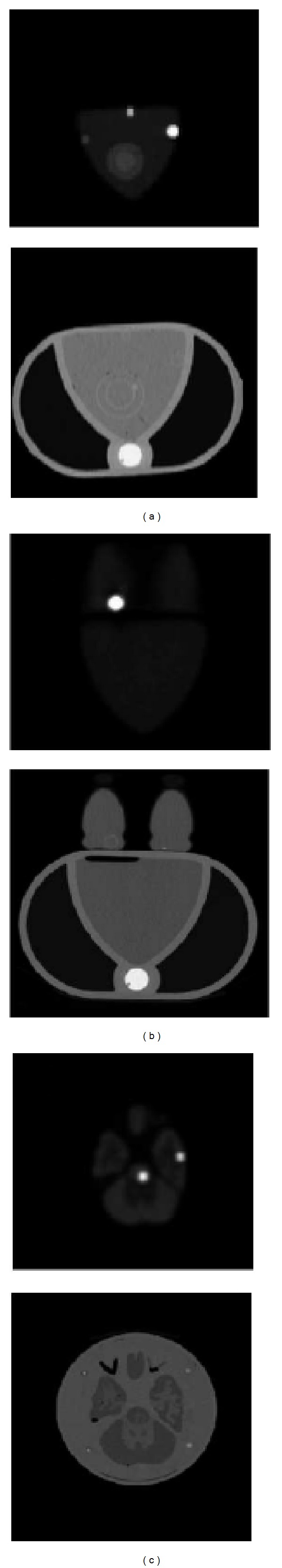
PET-CT images for (a) thorax phantom, (b) breast phantom, and (c) brain phantom.

Figure 4 shows PET-CT representative images of the oncological phantoms used for the validation of the PVE correction method. 

**Figure 5:**
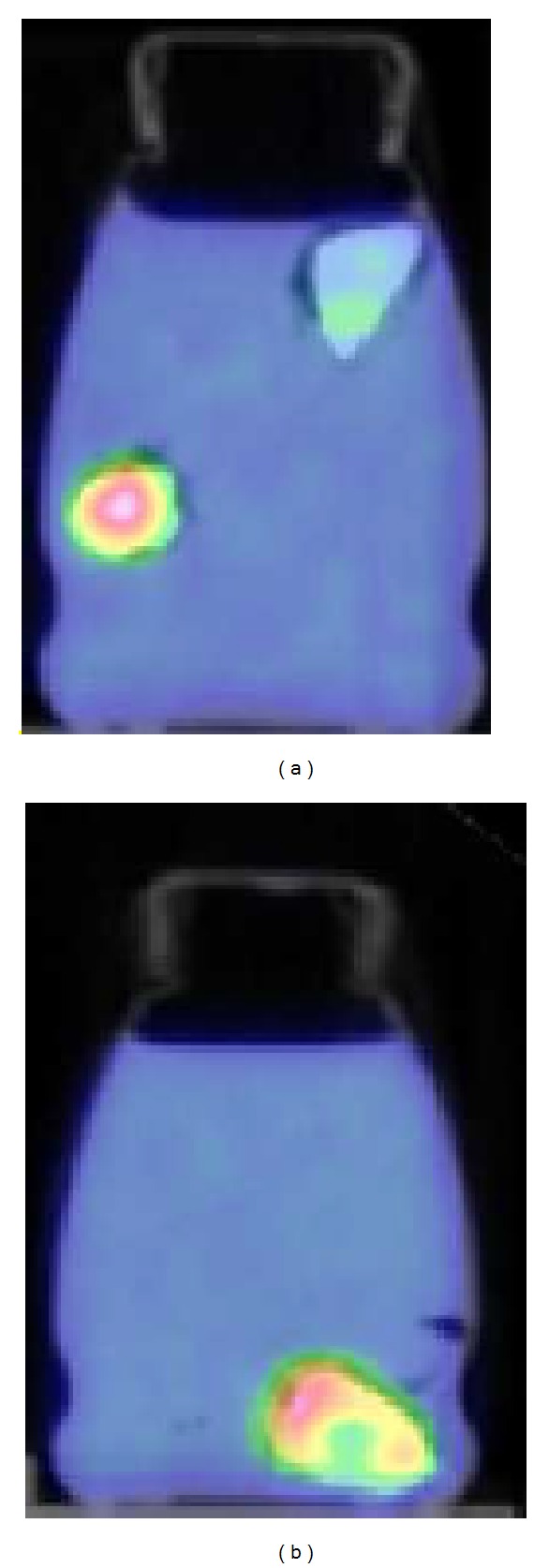
PET-CT images for the oncological nonspherical lesions (zeolites).

Figure 5 shows PET-CT representative images of the oncological nonuniform and nonhomogeneus lesions (the three zeolites) used for the estimation of bias of the PVE correction method for nonspherical lesions. The uniform uptake of the zeolite soaked in the radioactive solution for 15 minutes and the nonuniform uptake of the two zeolites soaked for few seconds can be observed.

*d*_60%_ of the spherical lesions ranged from 6 mm up to 12 mm and *L*/*B*_*m*_ ranged from 8 to 18.

Table 6 shows residual errors (%) after PVE correction, for all the lesions of the validation phantoms as percentage differences between the GS and PVE-corrected radioactivity concentration within the lesions. *C*_GS-sphere_, *C*_60%_, *L*/*B*_*m*_, and actual diameter are also presented.

For lesions with diameter > 1 cm, the PVE correction method was found with an accuracy > 91% in the thorax and breast. The method revealed an accuracy greater than 89% in the brain. 

For lesions with diameter < 1 cm, the residual error is of 24%, from an initial error of 70%. Thus, the method allows to recover 76% of radioactivity.

In case of zeolites, the PVE correction method confirms a good accuracy in the uniform lesion (% residual error < 17%). The method is not accurate for nonuniform lesions (zeolites with nonuniform uptake (% residual error > 30%)).

### 3.4. Feasibility of the PVE Correction Method

For all 149 lesions, it was possible to define the metabolic volume on PET images. 100% of lesions were found to have an *L*/*B*_*m*_ in the range of *L*/*B*_*m*_ measured from the spheres and lesion sphere-equivalent diameters in the range of sphere-diameters of RC curves. 

97% of lesions were found to have a spherical functional volume; 83% of lesions were found to have a uniform lesion uptake. 

Only for 25% of lesions, the lesion volume was visible on CT images. 

PVE correction was found to modify both the value of SUV and of SUV variations during patient followup. After PVE correction, SUV was found to be increased more than 25% in 31% of lesions with a percentage difference between PVE-affected SUV and PVE-corrected SUV up to 120%. SUV variations during followup were also found to be modified by PVE correction of >50% for 67% of lesions and up to 200%.

PVE correction was found to increase the statistical significance of statistical correlation tests (*P* changed significantly) between SUV and prognostic factors as histopathological indexes (histological grade, cell proliferation index, and therapy outcome indexes), allowing to identify a prognostic value of SUV for the considered cohort of oncological patients. As a consequence, SUV corrected with the proposed PVE was able to stratify different groups of patients.

Table 7 summarizes the main results of the impact of PVE correction on the considered correlation studies in the oncological patients.

PVE was also found to have an impact on the classification of patient response to treatment based on EORTC recommendations. Noteworthy, PVE correction changed the response classification of 3 of the 19 patients with bone metastasis (EORTC response classification: partial metabolic response, PMR; stable metabolic disease, SMD; progressive metabolic disease, PMD). In particular, one patient changed from PMR to SMD, one patient from SMD to PMD, and one patient from SMD to PMR. 

Table 8 summarizes the main results on the impact of PVE correction on the considered therapy response in the oncological patients. Applying PVC, the average SUV values increased more than 45%, proving the need for correction.

## 4. Discussion and Conclusions

Two aspects mainly characterise the proposed PVE correction method and differentiate it from other RC-based PVE correction procedures.

*(1) The Clinical Approach for the Design of PVE Correction*. The approach for the design of the PVE correction method moved from considering information from real clinical PET-CT studies, which is always available. The purpose of the work was the development of a PVE correction method allowing quantification of glucose metabolism in tumour cells with the primary objective to be easily implementable and usable in a clinical environment. Several studies showed that PET-detected oncological lesions are not always visible on CT images [27, 28] and this has also been confirmed by our nuclear medicine physicians. Thus, information which is always available is represented by data measurable on PET images, for instance, PET *L*/*B*_*m*_ and PET measured lesion volume. Following this consideration, our PVE correction method was based on RC factors derived from PET measurements of hot spheres in hot background, simulating lesions in body tissue under PET study. RC curves were thus obtained from PET *L*/*B*_*m*_ and from PET measured sphere volume, and not from actual *L*/*B* or from actual sphere volume, as in the case of all the rest of RC-based PVE correction methods. This strategy allows to overcome the problem of being aware of the actual *L*/*B* and the actual lesion volume.

*(2) The Technique for the PET Measurement of L*/*B*_*m*_ *and Lesion Metabolic Volume*. A technique allowing PET measurement of both lesion uptake (and thus PET *L*/*B*_*m*_) and lesion metabolic volume was developed, based on a technique of threshold isocontours. Such technique is not aimed at extracting the actual lesion metabolic volume but it is able to provide a PET measurement of a lesion metabolic volume (the “isocontour” volume) which is strongly dependant on the actual metabolic volume (the larger the actual metabolic volume, the “larger” the isocontour volume), however, being independent of the operator. The optimal volume, that is the volume defined by the optimal threshold, is the best metabolic volume matching the actual lesion volume and excluding at the same time the background uptake [30]. In this work, we present results of an optimal threshold relative to a 60% threshold. From the current literature [16], among the studied thresholds (in the range of 50–80%), the “isocontour” volume derived from a 60% threshold has been shown to represent a well compromise between a good sample of the lesion actual volume and a good sample of the lesion uptake, minimizing the possibility to include radioactivity background in the sample [36]. As assessed also by Krak et al. [30], a 50% threshold leads to include nontumour tissues, and this increases the possibility to include within the lesion normal high uptake in localized areas (e.g., liver, heart, and inflammatory tissues) that could be adjacent to the lesion. Furthermore, a threshold greater than 75% shows “less reproducibility” than lower thresholds (the difference in lesion metabolic volumes measured by PET at consecutive days is >50% for a threshold of 75% and <25% for a threshold of 60%, resp.)—both in terms of lesion metabolic volume and SUV [30]. Our results, relative to a threshold of 60%, show that the proposed PVE correction technique is accurate for lesion diameter > 1 cm, considering that previous studies on SUV reproducibility from oncological patients showed SUV percentage errors up to 17% [40].

The advantages of our approach are as follows.

*(A1) Consistency. *There is a full consistency between the direct procedure of obtaining RC from PET measurements with hot spheres in hot background and the inverse procedure that applies *F* = 1/RC factors for PVE correction of PET-detected oncological lesions. This allows for the clinical implementation of the PVE correction method to real oncological studies.

*(A2) Operator Independency*. The operator independency of the threshold technique for the PET measurement of quantitative parameters (PET *L*/*B*_*m*_ and PET measured “isocontour” volume) required by our PVE correction method guarantees reproducible measurements. Furthermore, the use of metabolic volumes defined by a threshold technique in clinical follow-up studies is suitable to show the effect of metabolic change due to therapy. This instead is not true for the CT detected anatomical volumes that may result unmodified at followup. As a result, our PVE correction method is more feasible for quantification of follow-up studies than alternative strategies based on actual lesion volume (e.g., [30]).

*(A3) Applicability*. The PVE correction method can be applied for any PET-CT scanner in a simple manner, given that it lies upon experimental measurements easy to be performed with a PET scanner and a standard phantom of easy availability. Anthropomorphic phantoms miming oncological lesions in specific regions of the human body could be used to extract RC factors more accurately for specific body regions (e.g., brain) or for a specific radiotracer (e.g., ^11^C-choline).

The disadvantages of our approach are as follows.

*(D1) Local Correction*. As for all RC-based PVE correction methods, our method applies PVE correction only at a regional level, on the PET images. This means that the lesion uptake is corrected using some information (PET *L*/*B*_*m*_ and PET measured lesion volume) of that particular region. As opposed to PVE correction methods which process PET images for the creation of PET corrected images (e.g., [20, 44, 45]), our method requires the correction to be applied separately to different lesions.

*(D2) Noise Dependency*. One of the drawbacks of the threshold technique for the PET measurement of lesion uptake and lesion metabolic volume is that the resulting defined region can be dependent on the noise present in the PET images. The threshold value for the radioactivity concentration (thus the corresponding isocontour) is dependent on the maximum value of the lesion uptake, being the threshold defined as a percentage of this value. Optimisation strategies based on smoothing or averaging techniques over the maximum could be applied [16] in order to reduce this effect.

*(D3) Lesion Roundness and Uniformity*. RC values have been obtained for hot spheres miming spherical and uniform lesions. This limits the application of the proposed PVE correction to oncological lesions which can be assumed to be spherical and with a uniform uptake. Preliminary results from our simulations on lesions with nonuniform uptake (zeolites) indicated that the PVE correction method is very sensitive to nonspherical and nonuniform lesions, while it can work well in nonspherical but uniform lesions, consistently with some results from Monte Carlo simulations proving the suitability of RC-based PVE correction for nonspherical lesions (e.g., [46, 47]). Considering our PET-CT clinical studies, we found that this occurs for a limited number of cases (96% of lesions were spherical and 80% with a uniform uptake). For those lesions that have hypometabolic characteristics (e.g., low grade tumour in the cerebral white matter), other PVE correction methods (e.g., based on image-guided segmentation or preprocessing) can be applied (e.g., [19, 20, 27, 48–51]). 

However, for lesions that cannot be approximated to spheres, our PVE correction approach should be used carefully and it needs optimization (e.g., new RC from nonspherical objects) as well as validation (e.g., with anthropomorphic phantoms including nonspherical objects). The same care in the use of the considered RC-based PVE correction must be applied to heterogeneous lesions. A recent study that focused on the impact of PVE correction on tumor heterogeneity suggests in this case the use of local image deconvolution approach with expectation maximization and spatially variant point spread function (e.g., [52]).

*(D4) Background Uniformity*. An important problem in practice is that the background is usually not uniform. High uptake in localized areas (e.g., liver, heart, and inflammatory tissues) could be present in regions adjacent to the lesion. The use of a single threshold to segment metabolic lesion volume, as proposed in our method, could include these normal tissues. In the latter case, manual intervention could be needed in order to exclude background tissues, thus making our method more observer dependent. 

We have developed, implemented, and assessed a method for PVE correction of oncological lesions in PET clinical studies, based on RC factors and PET *L*/*B*_*m*_ and PET measured lesion metabolic volume. 

Phantom measurements proved that PVE strongly affects lesion quantification (up to 70%) and needs to be corrected. Consistently with previous findings [26, 27, 53], we found this effect to be increasing when sphere volume and *L*/*B*_*m*_ decrease. 

Measured RC curves allowed PVE correction to be applied to lesions of diameter up to 40 mm and for PET *L*/*B*_*m*_ from 2 to 30, answering the need of PET quantification for a large variety of oncological lesions.

An operator independent technique was developed and optimised for the PET measurement of lesion uptake and of lesion metabolic volume. The technique is based on a threshold that defined an isocontour with respect to the maximum uptake on PET image. Such isocontour defines either the region of interest for the PET measurement of sphere/lesion uptake or the circle-equivalent section of a PET measured sphere/lesion spherical metabolic volume (isocontour volume).

Our residual errors obtained after the application of the PVE correction method to anthropomorphic oncological phantoms, compared with the errors on the measurement of SUV (12%-13%) obtained by Krak et al. [30], proved that our method is accurate (>89%) in clinical realistic conditions for lesion diameter > 1 cm and it is able to recover 76% of radioactivity for lesions diameter < 1 cm in a consistent way with the errors on the measurement of lesion metabolic volume (>23%) estimated by Krak et al. Other methods based on postreconstruction iterative techniques [44], iterative deconvolution [43], image segmentation [18], or multiresolution approach [20] implemented for PVE correction mainly in neurodegenerative diseases show an accuracy up to 98% for lesion diameter > 1 cm and up to 86% for lesion diameter < 1 cm. However, these methods require images to be processed by dedicated software and are more complex to be implemented in clinical routine than RC-based methods, as previously discussed ((*A3) Applicability*) and also commented by Soret et al. [16]. 

Patient studies showed that the proposed PVE correction method is suitable and feasible in a clinical environment. *L*/*B*_*m*_ and “optimal” isocontour volume at 60% threshold of the maximum were used to obtain proper RC in order to correct the PVE-affected SUV for all considered patient lesions. The quantitative analysis was performed under the guide of an expert nuclear medicine physician. We found that at least 80% of selected lesions met the requirements of roundness and uniformity for an accurate use of the proposed PVE correction method. As expected, only few lesions were clearly visible on CT images, confirming the need to define lesion volume from PET images. 

Considerations on SUV increase or decrease during patient followup as an effect of a therapy is beyond the purpose of this paper. However, our results suggest that the use of PVE correction can be fruitful in staging oncological disease and in monitoring oncological disease progression.

Our results suggest that the PVE correction has to be applied if SUV is used to stratify patients on the basis of an SUV cut-off value and/or to classify lesion metabolic response by means of SUV variations during followup. When SUV is considered for diagnostic purposes (i.e., an absolute cut-off value of SUV to differentiate benign from malignant tumor), the cutoff should be defined by accounting for PVE; otherwise it could be inappropriate.

In conclusion, in this work, we developed a method for PVE correction tailored for clinical application to PET-CT oncological studies. Our method overcomes the problem of considering actual *L*/*B* and actual lesion volume, being grounded in RC curves determined as functions of PET *L*/*B*_*m*_ and measured lesion volume, both estimated by an optimized and validated operator-independent technique. The proposed PVE correction method was applied to clinical oncological ^18^F-FDG PET-CT studies showing to have an impact on the metabolic assessment of lesions.

## Figures and Tables

**Table 1:** Characteristics and available data of patients.

Patient group	*N*	Purpose	Available data
Gastro	49	Tumor staging	^ 18^F-FDG PET-CT study (basal), tumor histotype (SRC, SC)
Breast	40	Tumor staging	^ 18^F-FDG PET-CT study (basal), Mib-1
Head-neck	19	Tumor staging	^ 18^F-FDG PET-CT study (basal), DFS
Skeleton	29	Therapy monitoring	Basal and follow-up ^18^F-FDG PET-CT studies

**Table 2:** Six spheres, one representative measurement: actual diameter, GS radioactivity concentration in the spheres and in the background and the derived *L*/*B*_GS_.

*d* (mm)	*C*_GS-sphere_ (MBq × mL^−1^)	*C*_GS-background_ (MBq × mL^−1^)	*L*/*B*_GS_
10	0.07844 ± 0.00666	0.01258 ± 0.000555	6.3 ± 0.65
13	0.07363 ± 0.00555	0.01258 ± 0.000555	5.9 ± 0.56
17	0.06475 ± 0.00222	0.01258 ± 0.000555	5.2 ± 0.35
23	0.06438 ± 0.00185	0.01258 ± 0.000555	5.2 ± 0.34
29	0.05550 ± 0.00111	0.01258 ± 0.000555	4.4 ± 0.27
37	0.05550 ± 0.00037	0.01258 ± 0.000555	4.4 ± 0.26

**Table 3:** Statistical analysis performed for the patient groups.

Patient group	Analysis
Gastro	Correlation between SUV and histological grade (Mann-Whitney test)
Breast	Correlation between SUV and Mib-1 (Mann-Whitney test)
Head-neck	Correlation between SUV and DFS (Log-rank test)
Skeleton	Classification of response to treatment (EORTC evaluation)

**Table 4:** Six spheres: percentage differences (%) between actual sphere diameter (d) and sphere diameter derived from “isocontour” volumes at 50, 60, 70, and 80%.

*d* (mm)	*d*_50%_ (mm)	% diff	*d*_60%_ (mm)	% diff	*d*_70%_ (mm)	% diff	d_80%_ (mm)	% diff
10	12	−20 ± 6.9	9	10 ± 1.8	7	30 ± 8.9	5	50 ± 17.4
13	12	7.7 ± 0.9	10	23.1 ± 2.0	8	38.5 ± 3.2	7	46.2 ± 5.1
17	16	5.9 ± 0.7	13	23.5 ± 0.9	12	29.4 ± 0.9	10	41.2 ± 1.5
23	19	17.4 ± 0.5	17	26.1 ± 0.8	15	34.8 ± 1.4	13	40.9 ± 2.5
29	27	6.9 ± 0.5	25	13.8 ± 0.8	23	20.7 ± 0.9	19	17.4 ± 1.0
37	34	8.1 ± 0.03	32	13.2 ± 0.1	30	18.9 ± 0.4	28	24.3 ± 1.1

**Table 5:** Six spheres, one representative measurement: % difference between GS radioactivity concentration and measured radioactivity concentration and the derived *L*/*B*_*m*_.

*d* (mm)	*C*_GS-sphere_ (MBq × mL^−1^)	*C*_60%_ (MBq × mL^−1^)	% diff	*L*/*B*_*m*_
10	0.078449 ± 0.00666	0.02331 ± 0.0037	70.3 ± 12.7	2.0 ± 0.4
13	0.07363 ± 0.00555	0.03774 ± 0.0074	42.5 ± 6.4	3.3 ± 0.5
17	0.06475 ± 0.00222	0.03959 ± 0.0037	39.0 ± 4.9	3.5 ± 0.4
23	0.06438 ± 0.00185	0.04033 ± 0.0074	37.4 ± 5.8	3.5 ± 0.6
29	0.5550 ± 0.00111	0.03330 ± 0.0037	40.0 ± 6.0	2.9 ± 0.4
37	0.5550 ± 0.00037	0.037774 ± 0.0037	31.9 ± 3.8	3.3 ± 0.4

**Table 6:** Validation phantoms: % residual errors after PVE correction.

Phantom	*d* (mm)	*C*_GS-sphere_ (MBq × mL^−1^)	*C*_60%_ (MBq × mL^−1^)	% res	*L*/*B*_*m*_
Thorax	9.8	0.8214	0.5883 ± 0.1036	24 ± 5.0	17.8
	12.3	0.3626	0.3293 ± 0.0333	9.8 ± 1.0	8.9
	12.3	0.6993	0.666 ± 0.0629	4.9 ± 0.5	16.8
	15.6	0.9065	0.8473 ± 0.0481	6.7 ± 0.4	30
	15.6	0.46028	0.45917 ± 0.06845	0.3 ± 0.04	9.2

Breast	9.8	0.0962	0.0777 ± 0.0074	16.6 ± 2.4	4.9
	12.3	0.1184	0.1073 ± 0.0148	9.3 ± 1.4	13.3
	15.6	0.2479	0.2590 ± 0.0148	−4.5 ± 0.3	8.3
	15.6	0.4884	0.4662 ± 0.0592	4.7 ± 0.8	20.3
	13.3*	0.0048*	0.0056 ± 0.00001*	−16.6 ± 2.9*	3.1*
	10.3**	0.0100**	0.0070 ± 0.00001**	30.0 ± 5.1**	2.8**
	9.9**	0.0128**	0.0049 ± 0.00007**	62.7 ± 11.1**	2.4**

Brain	9.8	0.5402	0.4070 ± 0.0666	24 ± 4.1	8.8
	12.3	0.4555	0.4033 ± 0.1184	11.4 ± 3.3	12.8
	12.3	0.4144	0.3663 ± 0.0703	11.2 ± 2.2	11.9
	15.6	0.3737	0.3552 ± 0.037	4.8 ± 0.5	14.2

*Represents zeolites with uniform uptake; **represents zeolites with nonuniform uptake.

**Table 7:** Oncological patients: results of SUV quantification with PVE correction on correlation studies between SUV and prognostic factors (*P* is the result of statistical tests).

Patient group	Lesion *d* (cm)	SUV (g/cc)	*P*	PVE-corrected SUV (g/cc)	*P* after PVE correction
Gastro	2.15 ± 1.17	3.27 ± 1.22 (SRC)	*P* > 0.05	5.57 ± 3.22 (SRC)	*P* < 0.05
	(0.99–6.25)	7.93 ± 5.01 (SC)		9.90 ± 1.91 (SC)	
Breast	1.57 ± 0.5	2.28 ± 1.02 (Mib+)	*P* > 0.05	4.52 ± 2.92 (Mib+)	*P* < 0.05
	(1.1–3.2)	7.64 ± 6.08 (Mib−)		9.30 ± 7.40 (Mib−)	
Head-neck	1.52 ± 0.5	<10.8 (lymph−)	*P* > 0.05	<13.3 (lymph−)	*P* < 0.05
		>10.8 (lymph+)		>13.3 (lymp+)	

**Table 8:** Oncological patients: results of SUV quantification with PVE correction on therapy response classification (EORTC). I means pretreatment SUV; II means posttreatment SUV.

Patient group	Lesion *d* (cm)	SUV (g/cc)	PVE-corrected SUV (g/cc)	SUV% difference
Skeleton	1.55 ± 0.5	4.7 ± 1.9 (I)	6.6 ± 2.3	46.4 ± 29.7
	(0.9–3.4)	4.2 ± 1.9 (II)	5.8 ± 2.6	45.9 ± 28.7
